# Validation of Antidiabetic and Antihyperlipidemic Effects of 80% Methanolic Extract of the *Lonchocarpus laxiflorus* Leaves in Streptozotocin-Induced Diabetic Swiss Albino Mice

**DOI:** 10.1155/2022/8411851

**Published:** 2022-12-28

**Authors:** Getaye Tessema Desta, Yared Andargie Ferede, Woretaw Sisay Zewdu, Baye Yrga Adugna, Tilaye Arega, Muluken Adela Alemu

**Affiliations:** ^1^School of Pharmacy, College of Medicine and Health Science, Debre Tabor University, Debre Tabor, Ethiopia; ^2^Department of Pharmacy, Bahir Dar Health Science College, Bahir Dar, Ethiopia

## Abstract

**Introduction:**

Diabetes mellitus (DM) is a chronic endocrine disorder that requires long-term treatment. In Ethiopian traditional medicine practice, plants have been provided with a vital role in fighting human and animal diseases since ancient times. The aqueous extract of *Lonchocarpus laxiflorus* (*L. laxiflorus*) leaves has been consumed for treating diabetes mellitus without confirming its safety and efficacy scientifically. This experiment aimed to evaluate the safety and antidiabetic efficacy of the leaf extract of *L. laxiflorus* in mice models.

**Methods:**

The crude extraction was conducted using a cold maceration technique and 80% methanol solvent. Normoglycemic, oral glucose-loaded, and streptozotocin-induced (STZ) diabetic models were employed. Male Swiss albino mice were randomly grouped into five categories( with six mice per group during normoglycemic, oral glucose-loadingtest as a negative control, positive control, and three treatment groups. In STZ-induced diabetic models, the groups include normal and diabetic negative control, diabetic positive control, and three diabetic treatment groups. The negative control groups received vehicles, the positive control received 5 mg/kg glibenclamide, and the treatment groups received the crude extract at 100, 200, and 400 mg/kg doses, respectively.

**Results:**

Up to 2000 mg/kg crude extract, neither signs of toxicity nor death were observed. In normoglycemic mice, there was a significant blood glucose reduction at 200 and 400 mg/kg doses starting from the 2^nd^ h post-administration. The oral glucose load showed a significant antihyperglycemic effect at 200 and 400 mg/kg of the crude extract and glibenclamide. In STZ-induced diabetic models, the 200, 400 mg/kg crude extract, and glibenclamide showed a significant antidiabetic activity and enhancement of a good serum lipid profile.

**Conclusion:**

This study confirmed that the leaf of *L. laxiflorus* was safe and possesses antidiabetic and antidyslipidemic activities.

## 1. Introduction

DM is a chronic metabolic disorder of carbohydrates, lipids, and proteins. It results from either the deficiency of insulin secretion secondary to pancreatic *β*-cells destruction or the development of insulin resistance for its action at insulin-sensitives peripheral tissues or from both [1]. The number of DM cases has increased year-by-year throughout the world. According to the 2019 International Diabetes Federation (IDF) report, the prevalence of DM was 9.3% and accounted for 463 million people. In 2019, 4.2 million adults (20–79 years old) deaths occurred as a result of DM and accounted for 11.3% of all causes of death [2, 3].

DM requires lifelong treatment to reduce the devastating problems of it. Currently available antidiabetic drugs mediated their pharmacologic action by enhancing insulin secretion, enhancing the sensitivity of insulin at the target tissue, reducing glucose absorption, delaying gastric emptying, and increasing satiety [4]. Though currently available antidiabetic drugs importantly controlled hyperglycemia, they caused serious side effects such as lactic acidosis, hypoglycemia, weight gain, and osteoporosis [5]. Beyond these serious side effects, they are not easy to access at an affordable price. As a consequence of this, scientists continued their search to discover a novel antidiabetic drug with a better safety profile, efficacy, and new mechanism of action development. Plants have played an important role in the management of human disease since immemorial time up to now. According to the World Health Organization (WHO), about 65 to 80% of people in the world depend on plants and plant remedy-based medicine for their primary health care keeping, particularly in rural areas of developing countries as a result of low side effects, convenience for use, easy accessibility with affordable price, and high cultural acceptance by the majority of people particularly developing countries. Besides use as a therapeutical means in traditional medical practice, plants are also used as a source for the development of new drugs. Based on the estimation of WHO, approximately, 25% of currently marketed pharmaceutical products originated from the plants that were used traditionally. For instance, metformin, a commonly available antidiabetic drug, was derived from *Galega officinalis* [6]. Nowadays, WHO gives a great emphasis, encouragement, recommendations, and promotions to incorporate herbal remedies in national health care programs of each country [7–9].

A huge number of medicinal plants have been used for the management of DM in Ethiopian folklore medicine practice. Among commonly mentioned medicinal plants for the treatment of DM, the leaf of *L. laxiflorus,* the seed of *Trigonella foenum-graecum L.*, the seed of *Linum usitatissimum* [10], the latex of Aloe vera, the fruit of *Persea americana,* the leaf of *Thymus schimperi*[11], the tuber of *Dioscorea alata L.* [11], and the leaf of *Hypoestes forskaoli* [12] were found. In Ethiopian folklore medicine practice, the leaf of *L. laxiflorus* is used for the treatment of DM after boiling in water, and then, the filtrate is taken orally at the time of use [10].

*L. laxiflorus* is categorized under the kingdom of *Plantae*, *Fabaceae* family, and class *Angiosperm*, and a species of *legume*. The plant can grow up to 5–6 m in height [13]. In Uganda, the roots and bark of the parts of the plant are available for the treatment of diarrhea and Buruli ulcers [14]. It is distributed in different parts of Africa including Ethiopia. In Ethiopia, it has been known by an Amharic name “Amer” [13]. In Ethiopian traditional medicine practice, the bark, leaves, and root parts of the plant are used for the treatment of cancer disease, and the leaf part is used for the treatment of DM. An *in vitro* study on the methanolic extract from the root of *L. laxiflorus* showed the antibacterial activity against *Staphylococcus aureus, Pseudomonas aeruginosa*, *and Escherichia coli.* A qualitative phytochemical screening test on the methanolic extract from the root of *L. laxiflorus* revealed the presence of flavonoids, alkaloids, saponins, tannins, phenols, and steroids [15].

The ethanolic leaf extract of the *Lonchocarpus cyanescens*, one of the plant species under the genus of *Lonchocarpus*, indicated *in vitro* antidiabetic activity on *α*-amylase (IC50: 3.69 mg/ml) and *α*-glucosidase (IC50 = 0.13 mg/ml) diabetes-related enzymes and hypoglycemic activity in starch-loaded rats [16]. There were no extensive experimental studies on the pharmacological activities of *L. laxiflorus*. The aim of this study was to evaluate the antidiabetic and antihyperlipidemic activity of the leaf extract of *L. laxiflorus* in a scientific manner.

## 2. Materials and Methods

### 2.1. Drugs and Chemicals

During this experiment, the following drugs and chemicals were used: absolute methanol (Re Agentchem), distilled water (EPharm), chloroform (Nice Laboratory Reagent), sulfuric acid (Thermo Fisher Scientific), benzene (Nice Laboratory Reagent), ferric chloride (Super-Tek), ammonium hydroxide (Rankem), Mayer's reagent (May and Baker), 40% glucose solution (Reyoung pharmaceuticals), glibenclamide (Glc; Cadila Pharmaceuticals), and streptozotocin (Fisco Research Laboratories). All of the chemicals were of analytical grade.

### 2.2. Collection of Plant Materials

The leaf of the plant was collected and the specimen was identified and authenticated by a botanist with the specimen number (LL021) and deposited for future reference. To remove the dirt and soil materials on the leaf, the freshly collected leaves were washed using tap water and dried at room temperature under shade for two weeks in the laboratory room. To enhance the rate and yield of extraction, the dried leaves were ground into a coarse powder. The powdered leaf was stored in a clean plastic container until it was subjected to the process of extraction.

### 2.3. Preparation of the Extract

The cold maceration technique and 80% methanol solvent were used during crude extraction. Coarsely powdered (500 g) leaves were macerated in 2500 ml of 80% methanol for consecutive three days in Erlenmeyer conical flasks. To facilitate the process of extraction and enhance, the yield of extraction, agitation, and string were employed frequently. After 72 h of maceration, the extract was filtered using a double-layer muslin cloth and for clarification, and Whatman No. 1 filter paper was also applied. Then, the marc was re-macerated twice using fresh 80% methanol in the same manner as the previous. The filtrates from each successive maceration were combined, and the methanol portion was removed from the filtrate by evaporation under vacuum in a rotary evaporator (Yamato, Japan) set at 40°C. The remained aqueous residue was removed after overnight deep freezing and then lyophilized through a lyophilizer. The extracts obtained from the three successive extraction process were combined and stored in an airtight bottle in the refrigerator until it was subjected to the experimental procedure. The fresh stock solution was prepared just before the actual experiment was carried out with 2% Tween 80 in distilled water. The extraction process was conducted based on the method explained in reference [17].

### 2.4. Experimental Animals

The age of 8–12 weeks and 20–30 g of body weight of healthy male Swiss albino mice were used for all antidiabetic models. The mice were purchased from Ethiopian Public Health Institute, Addis Ababa. The mice were housed in a clean metabolic cage that was bedded with a chip of wood in a standard condition (12 h light and 12 h dark cycle) until the experimental procedure run. The mice had free to access a standard commercial pellet diet, which was purchased from Ethiopian Public Health Institution (EPHI), Addis Ababa, Ethiopia, and water *ad libitum*. Before initiation of the experiment, the mice were acclimatized to the laboratory condition for seven days. The mice were handled throughout the experiment according to the international standard guideline set for the Care and Use of Laboratory Animals [18]. Ethical approval and permission were requested and gained from the Research and Ethics Committee of College of Medicine and Health Science, Debre Tabor University with the reference number Sop/345/22 on May 4, 2022.

### 2.5. Preliminary Phytochemical Screening

#### 2.5.1. Test for Alkaloids

About 0.2 g of the crude extract of the plant was dissolved with 2 ml of 1% HCl and heated gently. Then, two drops of Mayer's (potassium mercuric iodide) reagent was added to a side of the tube containing the acidic solution. The appearance of a yellow-colored precipitate designated the presence of alkaloids [19, 20].

#### 2.5.2. Test for Flavonoids

A few drops of ferric chloride were added to the crude extract of the plant. The formation and the appearance of the blackish-red precipitate indicated the presence of flavonoid metabolite in the leaf extract [19].

#### 2.5.3. Test for Tannins

About 0.5 g of the dried extract of the plant leaf was dissolved and added into a tube containing 20 ml of distilled water, and then, it was boiled in a test tube for an hour; then, the extract was filtered. Then, a few drops of 0.1% ferric chloride were added to the filtrate. The observation of the blue-black color indicated the presentence of tannin [21].

#### 2.5.4. Test for Terpenoids

About 2 ml of chloroform was mixed with 5 ml crude extract, and the mixture was evaporated in the water bath and then boiled with 3 ml of concentrated sulfuric acid (H_2_SO_4_). The appearance of a grey color showed the presence of terpenoids [22].

#### 2.5.5. Test for Steroids

About 500 mg of the crude extract of the plant was dissolved in 3 ml of chloroform, and then, the mixture was filtered. To this filtrate, concentrated H_2_SO_4_ was added. The formation of a reddish-brown color in the lower layer of chloroform indicated the presence of steroids.

#### 2.5.6. Detection of Phenols

The leaf extract of the plant was treated with 3 to 4 drops of ferric chloride solution. The formation of a bluish color indicated the existence of phenols in the leaf [23].

#### 2.5.7. Test for Saponins

About 50 mg of the leaf extract was suspended in distilled water up to 20 ml of the suspension was prepared. The suspension was shaken in a cylindrical graduate for 15 minutes. The formation of the two black cm layers of foam indicated the presence of saponins [23].

#### 2.5.8. Test for Anthraquinones

500 mg of the crude extract was boiled with 0.01 M HCl, and the extract was filtered while it was still hot. The filtrate was then shaken with 10 ml of benzene. The benzene layer was removed, and 5 ml of 10% ammonium hydroxide was added. The appearance of a violet, red, or pink color in the ammonia phase is positive for the presence of anthraquinones [21].

### 2.6. Acute Toxicity Study

The acute oral toxicity test was conducted using female mice. Since female mice are more sensitive than males to toxicity, only female mice were selected to evaluate the acute oral toxicity test. The test was conducted according to the limit test recommendation of the Organization for Economic Cooperation and Development (OECD) guidelines 425 [24]. Nulliparous, nonpregnant five healthy female mice of 8–12 weeks of age were used. They were randomly selected and acclimatized for five days before the actual test was conducted. The mice fasted from food with free access to water for 3 h. After administration of single dosage of 2000 mg/kg of the extract using orally via oral gavage, the mouse fasted for 1 h from food with free access to water. The mouse was carefully observed for any change in physiological activities every 30 minutes in the first 4 h of dosage and then for one day. After one day of follow-up, the remaining four female mice were tested in the same manner as the first mouse. Then, mice were observed daily for the manifestation of any loss of appetite, lacrimation, tremors, hair erection, diarrhea, mortality, and other signs of toxicity for a total of weeks.

### 2.7. Grouping and Dosing of Animals

In normoglycemic, oral glucose-loaded, and diabetic models, male mice were used. In normoglycemic, oral glucose-loaded models, the mice were grouped into five groups with six mice per group.

Group I-The negative control group and treated with vehicle (10 ml/kg 2% tween-80 in distilled water).

Group II-The positive control group and treated with the standard drug, glibenclamide 5 mg/kg.

Group III–V-Treatment groups were treated with 100, 200, and 400 mg/kg of the crude extract.

In the STZ-induced diabetic model (the repeated daily dose of the crude extract in STZ-induced diabetic mice), the mice were grouped into six groups (five diabetics groups and one none diabetic group) with six mice per group.

Group I-The diabetic negative control group received vehicle (10 ml/kg 2% Tween-80 in distilled water).

Group II-The positive diabetic control group received 5 mg/kg glibenclamide.

Groups III to V-Treatment groups were treated with 100, 200, and 400 mg/kg of the crude extract.

Group VI–The normal (nondiabetic) control group received vehicle (10 ml/kg 2% tween 80 in distilled water). The process of grouping and dosing was conducted according to the procedure [25].

### 2.8. Measurement of the Blood Glucose Level

The blood glucose was drawn from the vein of the mice's tail after cutting aseptically with a surgical blade. The BGL was measured using a glucometer. The measurement was carried out three times and the average (triplicated) value was taken. The percentage of BGL reduction was calculated according to the following formula [26].

(1) $$\begin{array}{c} \%\;BGLR=\frac{\;Gb}{\;Gp}\times 100\text{,} \end{array}$$

where BGLR = blood glucose level reduction, Gb = blood glucose level at 0 h (baseline blood glucose level), and Gp = blood glucose level after treatment.

### 2.9. Induction of Diabetes

To induce DM, STZ was applied. A 0.1 M STZ solution was prepared in sodium citrate buffer (pH at 4.5), and the freshly prepared solution of 150 mg/kg single dose of STZ was administered intraperitoneally (IP) to mice that were fasted for the overnight period (14 h). Since female mice were less sensitive to STZ, they were excluded from the experimental procedure [27]. After 30 minutes of STZ administration, the mice were given free access to standard pellet, food, and water. To avoid the death of mice from severe hypoglycemia secondary to a rapid release of insulin as a consequence of the destruction of pancreatic *β*-cell destruction, the mice were given 1 ml/kg glucose (5%) solution after 6 h administration of STZ and for the coming 24 h. After three days of STZ administration, the mice were screened for induction of DM. Mice with fast BGL >200 mg/dL were considered diabetic and selected to run the experiment. The method was conducted based on the procedure mentioned in reference [26].

### 2.10. Hypoglycemic Effect of the Crude Extract in Normoglycemic Mice

The normal male mice that were fasted overnight (14 h) from food with free access to water were grouped randomly into five groups (six mice per group) and received their respective treatment as mentioned in Grouping and Dosing. The BGL was measured just before (0 h as baseline) administration of each treatment, and then, after at 1^st^, 2^nd^, 4^th^, and 6^th^ h post-administration of the respective treatment [25].

### 2.11. Antihyperglycemic Effect of the Crude Extract in the Oral Glucose Tolerance Test

The overnight fasted (14 h) mice were grouped into five groups with six mice in each group and received their respective treatment as mentioned in Grouping and Dosing. The mice were administered 2 g/kg glucose solution orally (this matches with 10 *μ*l/g of fasted body mass in 20% glucose solution) after 30 minutes of administration of their respective treatment (the vehicle, the standard drug, and the crude extract). Then, the blood sample was taken from each mouse, and the BGL was measured just before loading of glucose solution (at 0 h as baseline), and at 30, 60, 90, and 120 minutes post-glucose load [25].

### 2.12. Antihyperglycemic Effect of the Crude Extract in Streptozotocin-Induced Diabetic Mice

The overnight fasted (14 h) mice were randomly classified into six classes with six mice per class as explained in Grouping and Dosing. The fasted BGL and the weight of the mice were measured just before (0 h as baseline) initiation of the respective treatment for each class and then at the 7^th^ and 14^th^ days of post-treatment of the respective treatment [25].

### 2.13. Assessment of Serum Lipid Profiles

After the end of the antidiabetic evaluation of the crude extract (on the 15^th^ day of the experiment), the mice were fasted overnight period (14 h) and were sacrificed in a humanitarian manner using 150 mg/kg pentobarbitone IP. Then, the blood sample was drawn from cardiac puncture using a sterile tube, and the blood samples were left at room temperature for 2 h before being centrifuged. The supernatant was removed immediately to produce serum samples. The serum lipid profiles that were evaluated include the total cholesterol (TC), triacylglycerol (TG), very low-density lipoprotein cholesterol (VLDL-C), low-density lipoprotein cholesterol (LDL-C), and high-density lipoprotein (HDL) using an automated chemistry analyzer [28].

### 2.14. Statistical Analysis

All statistical data were analyzed using the Statistical Package for Social Science (SPSS) version 24 and expressed as mean ± standard error of the mean (SEM) [29]. The significant difference between- and within group for each parameter was analyzed using a one-way analysis of variance (ANOVA) followed by Tukey's post-hoc multiple tests. There was a statical significance value when *p* value is less than 0.05.

## 3. Result

### 3.1. The Yield of Crude Extracts

The hydromethanolic extract of *L. laxiflorus* leaf gave a yield of 75.4 g (15.68%) of a dark red dry residue.

### 3.2. Preliminary Phytochemical Screening

The qualitative phytochemical screening of the crude extract illustrates the presence of alkaloids, flavonoids, phenols, tannins, terpenoids, and anthraquinones (Table 1).

### 3.3. Acute Oral Toxicity Study

After loading of 2 g/kg of a single dose of the crude extract of *L. laxiflorus* leaf, neither death nor any signs of toxicity such as a change in breathing rate, paw licking, shivering, restlessness, motor activity, and diarrhea were observed in any of the mice.

### 3.4. The Effect of the Crude Extract on Normoglycemic Mice

The hypoglycemic effect of the crude extract of the *L. laxiflorus* leaf is presented in Table 2.

As compared to the negative control group, there was a significant BGL reduction in mice receiving 200 (*p* < 0.05) and 400 mg/kg (*p* < 0.01) crude extract starting from the 2^nd^ h post-administration, while the standard drug (glibenclamide 5 mg/kg) showed a significant reduction of the BGL at 1^st^ h of post-administration. Mice that received a 100 mg/kg dose of the crude extract failed to show a significant BGL reduction relative to the negative control group throughout the experimental period.

Compared to the baseline BGL, there was a significant reduction in BGL started from the 2^nd^ h post-administration of 200 and 400 mg/kg doses of the crude extract of the leaf; however, mice that received 100 mg/kg dose did not show a significant BGL decrement. Mice that received the standard drug (glibenclamide 5 mg/kg) showed a significant BGL decrement at the beginning of the 2^nd^ h when compared to the baseline BGL.

### 3.5. Antihyperglycemic Effect of the Crude Extract in the Oral Glucose Tolerance Test

The outcome of the crude extract of *L. laxiflorus* leaf in the oral glucose tolerance test in normoglycemic mice is presented in Table 3. The loading of oral 2 g/kg of 40% glucose to normoglycemic mice showed a peak BGL within 1/2 h of post-glucose loading, and the maximum peak was achieved in mice that received the vehicle. The five treatment groups (the test extracts, distilled water, and glibenclamide treated groups) showed different patterns in decreasing the BGL from the climax level toward the baseline level. Initially, there was no significant difference in the BGL among mice that received the loading dose of glucose (*p* > 0.05).

In within-group analysis, there was a significant BGL reduction in mice receiving 200, 400 mg/kg crude extract and glibenclamide as compared to the baseline BGL (0 h) starting from the 1^st^ and 2^nd^ h post-loading of oral glucose. Mice received the crude extract of 100 mg/kg, and the vehicle did not show a significant BGL reduction as compared to the baseline BGL.

In between-groups analysis, there was a significant reduction in the BGL after post-treatments (½^th^, 1^st^, and 2^nd^ h) in mice treated with 5 mg/kg glibenclamide, 200, and 400 mg/kg of the crude extract as compared to mice treated with vehicle (10 ml/kg Tween 80 in DW) starting from 0.5 h of post-administration of treatment. While mice treated with 100 mg/kg crude extracts failed to show a significant BGL reduction in all posttreatment time relative to the negative control group.

### 3.6. The Effects of the Repeated Daily Doses of the Crude Extract in STZ-Induced Diabetic Mice

The daily repeated doses of 200 (*p*< 0.01), 400 mg/kg (^*p*^ < 0.001) of the crude extract, and 5 mg/kg dose of glibenclamide (^*p*^ < 0.001) revealed a significant reduction in BGL in diabetic mice as compared to BGL of the diabetes control group (diabetic mice that received 10 ml/kg 2% tween in DW) on the 7^th^ and the 14^th^ days. While, 100 mg/kg dose of the crude extract failed to show a significant reduction in the BGL relative to the diabetes control group on the 7^th^ and 14^th^ days of the treatment when compared to the negative diabetic control. The percentage of BGL reduction on the 7^th^ and 14^th^ days of 200 mg/kg of the crude extract treatment was 18.10% and 23.56%, while the 400 mg/kg was 24.49%, and 29.38%, respectively, from the baseline BGL. The percentage of blood glucose reduction on the 7^th^ and 14^th^ days by glibenclamide was 27.84% and 31.51%, which was closely related to 400 mg/kg of the crude extract.

In between-group analysis, there was a significant lowering effect in the BGL in diabetic mice receiving 200, 400 mg/kg of the crude extract, and 5 mg/kg of glibenclamide on the 7^th^ and 14^th^ days as compared to the BGL on zero days (the baseline BGL). The normal and diabetic mice treated with a vehicle (10 ml/kg of 2% tween in DW) did not show a significant reduction in the BGL as compared to the baseline BGL on the 7^th^ and the 14^th^ days. The effect of the daily dose of the crude extract in STZ induced is summarized in Table 4.

### 3.7. The Effect of the Crude Extract on the Serum Lipid Level of Diabetic Mice

The significance of the crude extract on the level of serum lipid profile is shown in Table 5. Relative to the nondiabetic mice that received the vehicle (10 ml/kg 2% Tween 80 in DW), there was a considerable dropping in the serum level of HDL-C, and an elevation of the level of TC, TG, VLDL-C, and LDL-C in STZ-induced diabetic mice that received the 100 mg/kg dose of the crude extract and the vehicle. When compared to the diabetic negative control (the diabetic mice received the 10 ml/kg 2% Tween 80 in DW), mice that received the 200, 400 mg/kg of the crude extract and 5 mg/kg glibenclamide illustrated a significant reduction in the serum level of TC, TG, VLDL-C, and LDL-C, and elevation of HDL-C, but 100 mg/kg dose of the crude extracted devoid to improve the serum lipid profile when compared to the negative diabetic control group.

## 4. Discussion

Diabetes mellitus is a chronic metabolic disease that is characterized by elevation of the plasma glucose levels (hyperglycemic) as a result of impairment of glucose utilization secondary to insufficient insulin secretion or insensitivity of insulin in the peripheral tissues. STZ is a well-known diabetogenic chemical for the induction of DM and is isolated from the bacterium *Streptomycin achromogenes*. It is a glucose analog that is selectively taken up by pancreatic *β*-cells using glucose transporter-2 (GLUT2). The destruction of *β*-cells secondary to STZ mediates by the nitrosourea-alkylating constituent and causes the damage of DNA through transferring of a methyl group from STZ to the DNA molecule and forming an alkylation and form a cross-linking on the strands of DNA. It also induced cytotoxicity in pancreatic cells by enhancing oxidative stress, modification of the cellular metabolism, and mitochondrial dysfunction [27, 30].

In this study, the leaf extract of *L. laxiflorus* (200 and 400 mg/kg) and glibenclamide showed a significant lowering activity in the overnight fasted BGL in normoglycemic mice. This indicated that the leaf of the *L. laxiflorus* possessed the ability to induce antihyperglycemic activity. The observed antihyperglycemic action post-administration of the crude extract might be that the crude extract might stimulate the uptake of glucose by peripheral tissue such as fat and muscle, block the process of gluconeogenesis and glycogenolysis, facilitate the storage and the utilization of glucose in different parts of the body, and stimulate the release of insulin from the *β*-cell of the pancreas [31, 32].

The hypoglycemic effect of this study is in line with the hypoglycemic effect of the leaf extracts of *Lonchocarpus cyanescens,* which is grouped under a similar genus to *L. laxiflorus,* as reported from the previously conducted research [16]. Glibenclamide, which is grouped under a class of sulfonylureas, produced a hypoglycemic effect by its ability to stimulate insulin secretion from pancreas beta–cells [33]. The hypoglycemic effects of medicinal plants are a consequence of their biologically active constituents (phytochemicals) such as flavonoids, tannins, phenols, terpenoids, saponins, and steroids as reported by previous researchers [34].

Flavonoids, which were detected in the leaf extract of *L. laxiflorus,* showed a hypoglycemic effect secondary to stimulation of insulin secretion [16].

The oral glucose tolerance test (OGTT) is used to assess the glucose utilization capability of the body as a source of energy, and it is considered “a golden standard” for the analysis of normal, impaired glucose tolerance and type 2 diabetes in clinical and research areas [35]. In this study, the maximum peak of BGL was attained at 1/2 h of post-oral glucose loading. This reflex that the oral glucose loading is capable to induce a physiological type of DM. After 1/2 h of administration of the crude extract and the standard antidiabetic drug to normoglycemic mice, a significant reduction of BGL was observed in the dose and time-dependent fashion at 200 and 400 mg/kg dose of the crude and glibenclamide 5 mg/kg as compared to normoglycemic mice that received the vehicle. There was a significant reduction in the BGL 2 h post-administration of the crude extract of 200, 400 mg/kg and glibenclamide 5 mg/kg relative to the BGL reduction at 1/2 h. The observed BGL reduction suggested that the leaf of the plant has the potential to enhance the proper utilization of the body's glucose. The postprandial BGL reduction ability of the crude extract may be attributed to the hindrance of intestinal glucose absorption, block of hepatic glucose production, and stimulation of glucose uptake by the skeletal muscle and adipose tissue [36, 37]. In this study, the lower dose (100 mg/kg) of the crude extract could not show a significant impact on postprandial BGL. The possible reason might be the accommodation of a low amount and types of phytochemicals responsible for the antihyperglycemic activity of plant extracts.

The crude extract of the 200 and 400 mg/kg showed a significant antidiabetic activity as compared to diabetic mice that received a vehicle. The maximum antidiabetic activity on the 7^th^ and the 14^th^ days by 400 mg/kg dose of the crude extract, respectively, were 20.93 and 29.38%, which are closely related to the reference drug glibenclamide (22.49 and 30.44%) while 200 mg/kg dose. On the other hand, the lower dose of the crude extract was unable to show a significant lowering of BGL. The outcome of this finding indicated that the crude extract possessed antihyperglycemic potential, and this was in a dose- and time-dependent fashion. The antidiabetic activity of this find was in line with the antidiabetic finding from the extract of *Aloe pulcherrima* leaf latex in mice [38]. The likely antidiabetic mechanism action of the crude extract may be the restoration of pancreatic Beta cells, blocking of intestinal glucose absorption, stimulation of glucose utilization and uptake by peripheral tissue, inhibition of glucagon secretion, enhancing the hepatic production of glycogen, and stimulation of insulin secretion from the existing beta-cells of pancreas. The antidiabetic action of the plant extract is attributed to the presence of phytochemicals [39, 40]. Alkaloid isolate from plant extract enhances the uptake of glucose in the peripheral tissue [41].

The abnormality of serum lipid profile is among the factor associated with diabetic and prediabetes patients, which are characterized by the increasing serum level of TG, VLDL-C, and LDL- C and decreasing HDL-C [42]. Numerous medicinal plants have been studied for their hypolipidemic activity [43, 44]. In this study, 200 and 400 mg/kg doses of the crude extract of the *L. laxiflorus* illustrated a significant reduction in the serum level of TC, TG, VLDL-C, and LDL- C, and elevation of HDL-C when compared to the diabetic negative control (the diabetic mice received the 10 ml/kg 2% Tween 80 in DW), but the 100 mg/kg dose of the crude extracted devoid to improve the serum lipid profile. The finding of this study was in line with the finding of reference [45]. The hypolipidemic activity of plant-based therapy may be mediated by their bioactive substances (phytochemicals) such as saponins, alkaloids, and flavonoids [46], which were isolated from lotus, illustrating hypolipidemic activity in diabetic mice secondary to STZ administration [47]. Alkaloids separated from the extract of *Rhizoma Coptidis* showed antihyperglycemic and antihyperlipidemic [48]. These phytochemicals mediated their lipid-lowering activity through augmentation of fecal cholesterol elimination, the embarrassment of lipid absorption on the intestinal lumen, and inhibition of hepatic lipids synthesis.

Saponins separated from *Phaseolus vulgaris* L seed, which were also detected in the crude extract of *L. laxiflorus* leaf, showed a significant inhibitory activity of intestinal cholesterol absorption by forming a complex with cholesterol [49, 50]. Pancreatic lipase is an important enzyme that is responsible for the metabolism of TG. Tannin, which was detected in this crude extract, illustrated an inhibitory activity on pancreatic lipase [51].

## 5. Conclusion

This study confirmed that the crude extract of *L. laxiflorus* leaves possessed antidiabetic and hypolipidemia activities with a good margin of safety. We recommended to conduct further studies on the mechanism of action and the identification of the responsible phytochemical constituents.

## Figures and Tables

**Table 1:** The qualitative preliminary phytochemical screening test of the crude extract.

Screened physiochemical constitutes	Result of the screening
Alkaloids	Present
Flavonoids	Present
Tannins	Present
Terpenoids	Present
Steroids	Absent
Phenols	Present
Saponins	Absent
Anthraquinones	Present

**Table 2:** The hypoglycemic effect of the crude extract in normoglycemic mice.

*Blood glucose level (mg/dl)*					
Dose	0 h	1 h	2 h	4 h	6 h
NC	94.67 ± 1.18	90.32 ± 3.03	87.43 ± 3.29	84.94 ± 1.71	83.26 ± 1.72
GLC 5 mg/kg	93.32 ± 3.15	70.65 ± 3.03^a3^	69.4 ± 2.21^a3b2*β*3^	61.32 ± 1.71^a3b3*β*3^	54.72 ± 2.95^a3b3*β*2^
CE100 mg/kg	93.53 ± 0.94	89.21 ± 1.74	86.05 ± 1.8	82.40 ± 0.67	78.96 ± 1.88
CE200 mg/kg	92.79 ± 2.60	85.65 ± 3.01	80.49 ± 2.400^a1*β*1^	74.34 ± 1.28^a2*β*2^	64.66 ± 1.70^a3*β*3^
CE400 mg/kg	91.37 ± 1.79	82.08 ± 1.00	75.76 ± 1.4^a2*β*2^	68.94 ± 1.53^a3*β*3^	60.57 ± 1.86^a3*β*3^

Each value represents as mean ± SEM; *n* = 6 for each treatment, ^a^compared to the negative control, ^b^compared to 100 mg/kg, and ^*β*^compared to baseline blood glucose level. ^1^^*p*^ < 0.05, ^2^^*p*^ < 0.01, and ^3^^*p*^ < 0.001. NC = negative control and received 2% tween 80 indistilled water, GLC = glibenclamide, CE = crude extract.

**Table 3:** The effect of the crude extract on oral glucose loaded mice.

*Blood glucose level (mg/dl) at different time intervals*				
Dose (mg/kg)	0 h	0.5 h	1 h	2 h
NC	87.39 ± 2.07	164.00 ± 0.95	158.48 ± 1.93	153.03 ± 3.33
CE 100	92.59 ± 1.46	161.04 ± 1.40	151.38 ± 1.74	1487.51 ± 2.07
CE 200	94.28 ± 1.13	149.92 ± 2.48^a2^	135.18 ± 2.08^a3*β*3^	105.86 ± 2.27^a3b2*β*3^
CE 400	92.80 ± 1.87	142.66 ± 1.77^a3b3c2^	105.47 ± 3.89^a3b3*β*3^	93.99 ± 1.30^a3b3c1*β*3^
GLC 5	89.62 ± 1.92	138.40 ± 1.20^a3b3c2^	100.62 ± 4.21^a3b1*β*3^	89.82 ± 2.17^a3b3c3*β*3^

Each value represents as mean ± SEM; *n* = 6 for each treatment. ^a^Compared to the negative control, ^b^compared to 100 mg/kg, ^c^compared to 200 mg/kg, ^*β*^compared to baseline blood glucose level. ^1^^*p*^ < 0.05, ^2^^*p*^ < 0.01, and ^3^^*p*^ < 0.001. NC = negative control received 2% Tween in distilled water, and GLC = glibenclamide, CE = crude extract.

**Table 4:** The effect of the daily repeated dose of the crude extract in STZ-induced diabetic mice.

*Fasting BGL (mg/dl)*				*% of BGL reduction*	
Dose	Day 0	Day 7	Day 14	7^th^ day	14^th^ day
DNC	281.01 ± 1.25	278.67 ± 2.35	276.72 ± 1.25	0.83	1.53
GCL 5 mg/kg	279.78 ± 2.7	216.89 ± 2.96^*β*3a3b3^	194.61 ± 2.86^*β*3a3b3c3^	22.49	30.44
CE100 mg/kg	278.84 ± 2.33	254.15 ± 1.43	250.05 ± 1.11	8.85	10.32
CE200 mg/kg	277.35 ± 2.86	237.16 ± 1.57^*β*3a2b2^	212.02 ± 2.05^*β*3a3b3^	14.49	23.56
CE400 mg/kg	280.67 ± 1.97	221.93 ± 1.83^*β*3a3b3^	198.21 ± 3.36^*β*3a3b3c2^	20.93	29.38
NNC	94.43 ± 0.98	92.49 ± 1.77	89.76 ± 2.25	2.05	4.94

Each value represents mean ± SEM; *n* = 6 for each treatment^,^. ^*β*^Compared to day zero (baseline blood glucose level); ^a^compared to diabetes control; ^b^compared to 100 mg/kg, ^c^compared to 200 mg/kg, ^1^^*p*^ < 0.05, ^2^^*p*^ < 0.01, and ^3^^*p*^ < 0.001. BGL = blood glucose level, CE = crude extract, DNC = diabetes negative control received 10 ml/kg 2% tween 80 in distilled water GLC = glibenclamide, NNC = normal negative control received 10 ml/kg 2% tween 80 in distilled water.

**Table 5:** The effect of repeated daily dose of the crude extract on the serum lipid level of diabetic mice.

Serum lipid level (mg/dl)					
Dose	TC	TG	LDL-C	VLDL-C	HDL-C
DNC	192.41 ± 1.60^b3^	155.69 ± 2.84	102.33 ± 3.34^b3^	35.26 ± 1.49^b3^	25.73 ± 1.82
GCL 5 mg/kg	118.12 ± 4.14^a3^	83.59 ± 1.92^a3^	48.77 ± 1.07^a3^	21.13 ± 1.01^a3^	44.65 ± 1.31^a3^
CE100 mg/kg	168.17 ± 3.92^b3^	149.62 ± 3.11^b3^	90.37 ± 2.38^b3^	38.03 ± 0.41^b3^	30.67 ± 1.54^b3^
CE200 mg/kg	128.17 ± 1.18^a3^	89.31 ± 3.18^a3^	51.53 ± 1.56^a3^	25.68 ± 0.57^a2^	36.31 ± 1.02^a3^
CE 400 mg/kg	121.04 ± 0.69^a3^	86.59 ± 1.54^a3^	48.73 ± 1.53^a3^	23.51 ± 0.62^a3^	39.58 ± 0.57^a3^
NNC	115.43 ± 3.25^a3^	78.75 ± 2.33^a3^	41.08 ± 1.05^a3^	19.93 ± 0.42^a3^	45.35 ± 1.23^a3^

Each value represents mean ± SEM (*n* = 6 mice in each group) and analyzed by one-way ANOVA followed by Post Hoc Tukey's test; ^a^compared to the diabetic control, ^b^compared to the normal control; ^1^^*p*^ < 0.05, ^2^^*p*^ < 0.01, and ^3^^*p*^ < 0.001. TC = Total cholesterol, TG = triacylglycerol (TG), VLDL-C = verylow-density lipoprotein cholesterol, LDL-C = low-density lipoprotein cholesterol, HDL-C = high-density lipoprotein cholesterol, CE = crude extract, DNC = diabetes negative control that received 10 ml/kg 2% tween 80 in distilled water GLC = glibenclamide, NNC = normal negative control that received 10 ml/kg 2% tween 80 in distilled water.

## Data Availability

All the data that were used throughout the experiment procedure are available from the corresponding author and offered upon reasonable request.

## Abbreviations

BGL: Blood glucose level
DM: Diabetes mellitus
GLC: Glibenclamide
*L. laxiflorus*: *Lonchocarpus laxiflorus*
OGTT: Oral glucose tolerance test
STZ: Streptozotocin.
